# Role of Impaired Astrocyte Gap Junction Coupling in Epileptogenesis

**DOI:** 10.3390/cells12121669

**Published:** 2023-06-20

**Authors:** Peter Bedner, Christian Steinhäuser

**Affiliations:** Institute of Cellular Neurosciences, Medical Faculty, University of Bonn, Venusberg-Campus 1, 53127 Bonn, Germany

**Keywords:** astrocyte, gap junction coupling, connexin 43, connexin 30, hippocampus, epilepsy, epileptogenesis, temporal lobe epilepsy

## Abstract

The gap-junction-coupled astroglial network plays a central role in the regulation of neuronal activity and synchronisation, but its involvement in the pathogenesis of neuronal diseases is not yet understood. Here, we present the current state of knowledge about the impact of impaired glial coupling in the development and progression of epilepsy and discuss whether astrocytes represent alternative therapeutic targets. We focus mainly on temporal lobe epilepsy (TLE), which is the most common form of epilepsy in adults and is characterised by high therapy resistance. Functional data from TLE patients and corresponding experimental models point to a complete loss of astrocytic coupling, but preservation of the gap junction forming proteins connexin43 and connexin30 in hippocampal sclerosis. Several studies further indicate that astrocyte uncoupling is a causal event in the initiation of TLE, as it occurs very early in epileptogenesis, clearly preceding dysfunctional changes in neurons. However, more research is needed to fully understand the role of gap junction channels in epilepsy and to develop safe and effective therapeutic strategies targeting astrocytes.

## 1. Introduction

Epilepsy is a disease of the brain that affects about 1% of the world’s population and is characterised by the occurrence of recurrent unprovoked seizures [1,2]. Although more than twenty antiepileptic drugs (AEDs) are currently on the market, seizures cannot be fully controlled in about one-third of patients. Furthermore, available AEDs merely provide symptomatic treatment but are not able to prevent or reverse the development of the disorder [3]. These drugs act primarily on neurons through modulating voltage-activated ion channels, enhancing GABAergic inhibition or attenuating excitatory neurotransmission [3,4]. However, as the efficacy and tolerability of AEDs have not substantially improved in recent decades, and drugs with disease-modifying (antiepileptogenic) effects are still not available, this neurocentric strategy in the development of AEDs is increasingly being challenged. Indeed, it seems plausible that not only defective neurons but also a disruption of the control mechanisms that regulate neuronal excitability and synaptic transmission can cause epilepsy [5,6]. Astrocytes not only have crucial homeostatic and supportive functions, such as the supply of energy substrates to neurons, the control of extracellular ion and water homeostasis, the clearance of neurotransmitters from the synapse or the regulation of blood–brain barrier (BBB) permeability, but also actively modulate synaptic transmission and neuronal plasticity through the release of gliotransmitters [5,7]. Synapses interact with every physical component of an astrocyte, including endfeet and soma. An important prerequisite for the implementation and/or efficiency of most of these regulatory functions is the fact that astrocytes are extensively electrically and metabolically interconnected via low-resistance aqueous channels termed gap junctions (GJs). This leads to the formation of large syncytium-like functional networks that overlap with neuronal synaptic networks and enable their coordinated regulation and synchronisation [8,9]. Astrocytic gap junctions consist mainly of the channel proteins connexin43 (Cx43) and connexin30 (Cx30), whose relative expression levels vary considerably across developmental stages and brain regions [9,10,11]. In adult mouse and human hippocampi, astrocyte coupling appears to be predominately mediated by Cx43 [10,12,13], while mouse thalamic astrocytes couple mainly through Cx30 [11]. The two astrocytic Cx isoforms possess different biophysical properties (unitary conductance, selectivity, transjunctional voltage sensitivity and gating properties) and are differentially regulated [14,15]. The gating properties of Cx43-based channels as well as the trafficking, assembly and degradation of Cx43 proteins are tightly regulated by phosphorylation [16]. In contrast, there are no reports on Cx30 phosphorylation. Astrocytic Cxs can form in addition to homotypic GJ channels also functional heterotypic channels with each other and with oligodendrocytic Cxs [11,17,18]. In addition, under certain (primarily pathological) conditions, undocked Cx hemichannels (HCs) might also function as transmembrane channels, allowing the bidirectional exchange of ions and small molecules between the cytoplasm and the extracellular space [8,19]. Finally, there is growing evidence that Cx proteins play an important role in several other fundamental processes, including adhesion, migration, cellular life cycle, neurogenesis, cell death and signal transduction [8,20,21].

## 2. Role of Glial Cx Channels in Epilepsy

In view of the essential role of the astrocyte network for neuronal function and brain homeostasis, it is not surprising that various neurological disorders including stroke, migraine, gliomas, Alzheimer’s disease and epilepsy have been linked to alterations in astrocyte coupling [8]. The role of astrocytic network disturbances in the development and progression of epilepsy is not yet conclusively understood, but there is growing evidence that they are fundamentally involved [5,22,23,24,25]. In principle, disruption of GJ coupling between astrocytes can affect neuronal excitability and synchronisation in many ways. An important epilepsy-promoting functional consequence of such a disruption would be the reduced astrocytic capacity to buffer elevated extracellular K^+^ concentrations ([K^+^]_o_) during high neuronal activity. In the process known as spatial K^+^ buffering, the excess neuronally released [K^+^]_o_ is passively taken up by astrocytes via Kir4.1 channels, distributed in the GJ-coupled network and released at sites of lower [K^+^]_o_ [26]. This process, which is driven by the difference between the local K^+^ equilibrium potential and the more negative membrane potential of the glial syncytium, cannot be sustained in the absence of coupling, resulting in stronger local [K^+^]_o_ increases and consequently in a more pronounced neuronal depolarisation and enhanced neuronal excitability [26,27,28,29,30]. In addition to K^+^, the redistribution of Na^+^ ions through the GJ-coupled astrocytic network might also play an important role in preventing neuronal hyperexcitability. In fact, astrocytic plasmalemmal glutamate transporters are mainly responsible for the removal of excess glutamate during synaptic activity, a process driven by the Na^+^ gradient and involving massive co-uptake of the ion [31,32,33]. Reduced GJ coupling could promote accumulation of cytosolic Na^+^ in astrocytes, resulting in a reduced driving force for uptake of synaptically released glutamate and thus increased excitatory neurotransmission [34]. Furthermore, such increased cytosolic Na^+^ can trigger the reversal of the Na^+^/Ca^2+^ exchanger, resulting in effective astrocytic Ca^2+^ uptake and therefore defective Ca^2+^-dependent processes such as aberrant gliotransmitter release, which in turn also affects network excitability [31,33,35,36]. Finally, cytosolic K^+^ and Na^+^ accumulation enhances water influx and thus the extent of activity-dependent astrocytic swelling. The resulting reduction in extracellular space volume further increases the concentration of extracellular ions and neurotransmitters, exacerbating the seizure-promoting effect of impaired spatial K^+^ and glutamate buffering [22,37]. Experimental confirmation for these theoretical considerations is provided via experiments on brain slices from transgenic mice with coupling-deficient astrocytes or after pharmacological disruption of GJ communication. Uncoupling, in fact, resulted in impaired spatial K^+^ and Na^+^ buffering [29,30,32,38,39], reduced astrocytic glutamate clearance [37] and hypertrophic astrocytes [37,40]. Together, these findings point to an anti-epileptic function of the astroglial network. On the other hand, it was shown that the astrocytic network is crucial for the effective supply of energetic metabolites from blood vessels to sites of high neuronal activity [41,42]. It can be assumed that this function of the network has less effect on the initiation than on the maintenance of seizure activity. Thus, an acute reduction of astrocytic GJ coupling may include rapid seizure-promoting consequences due to reduced K^+^ and glutamate buffering but delayed seizure-suppressing effects due to insufficient energy supply [22,23,31]. Reduced coupling may also restrict the propagation of Na^+^ and Ca^2+^ through the astrocyte network and thus its ability to synchronise large populations of neurons. This effect may counteract the hypersynchronous neuronal firing that characterises epilepsy [5,22,31,43].

In addition to intercellular GJ channels, functional Cx HCs have also been reported to play an important role in the pathology of various diseases, including epilepsy, al-though it is unclear how cells can maintain their integrity upon opening of such large non-specific pores [8,44]. In the healthy brain, Cx HCs do not appear to have a significant open probability, but this increases in response to various stress conditions such as metabolic inhibition, hypoxia/ischemia, inflammation, strong depolarisation or altered intra- and extracellular Ca^2+^ concentrations [8,19]. In epilepsy, opened HCs were proposed to release neuroactive molecules such as glutamate, ATP and/or D-serine, which in turn enhance neuronal excitability and synchronisation. Accordingly, Cx HC activation has been proposed to have pro-epileptic effects [8,22,45,46,47].

**Figure 1:**
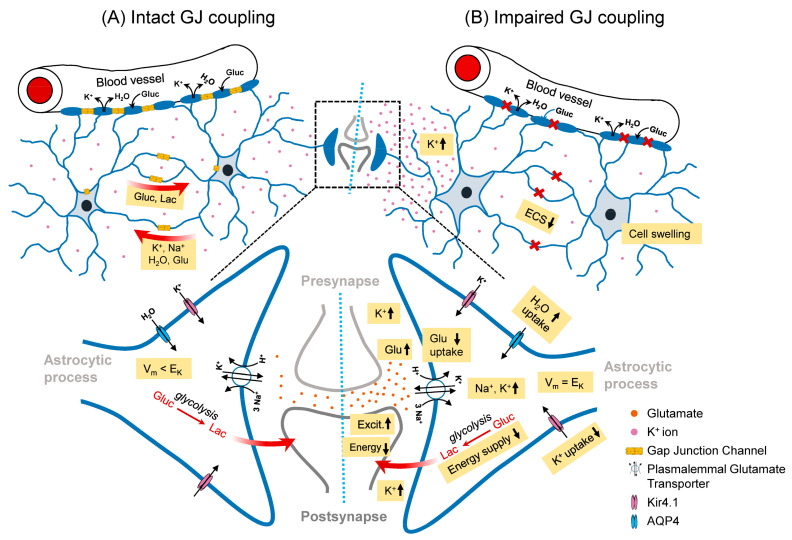
Schematic illustration of the role of the GJ-coupled astrocytic network in the healthy brain (left) and consequences of its disconnection (right) on astrocyte homeostatic functions and neuronal activity. (**A**) In the intact astrocytic network, excessive extracellular K^+^ released during high neuronal activity is taken up by astrocytes, redistributed through the astrocytic network and released at regions of lower extracellular K^+^ concentrations. This process is energy-independent, as the uptake and release of K^+^ through Kir4.1 channels are driven by the difference between the dynamic local K^+^ equilibrium potential (E_K_) and a relatively constant syncytial voltage (V_m_). Astrocytic synaptic glutamate (Glu) buffering leads to massive Na^+^ co-uptake, which is distributed in the astrocytic network. Astrocytic GJ coupling mediates activity-dependent trafficking of glucose (Gluc) and its metabolic product lactate (Lac) from blood vessels to sites of high energy demand. (**B**) In the absence of GJ coupling, an increased extracellular K^+^ concentration leads to depolarisation of nearby astrocytes (V_m_ = E_K_), resulting in cessation of K^+^ uptake and re-distribution through spatial buffering. This leads to increased/prolonged extracellular K^+^ transients and increased/prolonged neuronal depolarisation, lowering the threshold for seizure generation. Loss of coupling also leads to intracellular accumulation of Na^+^ taken up together with glutamate. Na^+^ accumulation and depolarisation triggered by elevated extracellular K^+^ reduce the driving force for glutamate uptake, resulting in a seizure-promoting prolongation of excitatory synapse activation. In addition, intracellular accumulation of K^+^ and Na^+^ trigger astrocytic water uptake through aquaporin-4 (AQP4) channels and a reduction of the extracellular space (ECS) volume due to astrocytic swelling, which in turn further increases the concentration of extracellular ions and neurotransmitters, exacerbating the seizure-promoting effect of impaired K^+^ and glutamate buffering. On the other hand, uncoupled astrocytes are less effective at providing energy substrates, which counteracts neuronal hyperactivity.

Finally, non-channel functions might also be involved in the pathogenesis of epilepsy. For example, Cx43 was shown to alter the expression and dose–response curve of glial purinergic P2Y_1_ receptors even in the absence of functional channels [8,48]. Due to its involvement in glial Ca^2+^ mobilisation and the propagation of astrocytic Ca^2+^ waves, the metabotropic P2Y1 receptor has been attributed an important role in epilepsy [49,50]. Another example of an epilepsy-relevant non-channel function of Cx proteins was provided by Pannasch and colleagues, who showed that Cx30 proteins control the intrusion of astroglial processes into the synaptic cleft, thereby indirectly modulating the efficiency of synaptic glutamate clearance and, thus, the strength of excitatory synaptic transmission [51] (Figure 1).

## 3. Connexin Expression and GJ Coupling in Human Epilepsy

The most relevant information about astrocytic changes in human epilepsy has been gained from epileptogenic brain tissue specimens surgically resected from patients with drug-resistant temporal lobe epilepsy (TLE). Several studies compared epilepsy-related changes in Cx expression, for example in surgical specimens from TLE patients with (HS) vs. without hippocampal sclerosis (non-HS), or in samples from epilepsy patients vs. non-epileptic patients [12,52,53,54,55,56,57,58]. Except for one study [56], all found upregulation of Cx43 transcripts or protein in epileptic or sclerotic tissue. A detailed analysis was carried out by Deshpande and co-workers, who compared not only whole cell protein levels of the two astrocytic Cx isoforms but also the plasma membrane-associated protein fractions, subcellular distribution and phosphorylation status of Cx43 between sclerotic and non-sclerotic human tissue from TLE patients [12]. Neither total nor membrane-associated Cx30 protein levels nor subcellular distribution of Cx30 differed between both conditions. Interestingly, in HS, increased Cx43 protein levels were found only in the total protein fraction but not in the plasma membrane fraction. Importantly, the authors also reported a pronounced subcellular redistribution towards the perivascular endfeet as well as an altered phosphorylation pattern of Cx43 in HS, a finding suggestive of altered astrocytic coupling [12]. Information on functional astrocytic changes in human epileptic tissue is sparse due to the limited availability of live tissue, the technical difficulties of recording from them and the lack of healthy control tissue. The first evidence of a disturbance in the ability of astrocytes to spatially buffer K^+^ in human TLE with HS was gained more than 2 decades ago, although it remained unclear whether changes in GJ coupling contributed to the phenomenon [59,60,61,62]. Characterisation of functional GJ coupling between human astrocytes in the sclerotic and non-sclerotic hippocampus was performed several years later via tracer diffusion analysis [24]. Intriguingly, in non-HS control-like tissue (obtained from patients with lesion-associated TLE, [62,63]) the extent of astrocyte coupling was in the same range as in the adult rodent hippocampus, indicating that GJ coupling has no influence on the pathogenesis of this type of epilepsy [13,24]. In contrast, complete loss of bona fide astrocytes and GJ coupling was found in hippocampal slices from TLE-HS [24]. Together, these data suggest that Cx proteins are abundantly expressed in the sclerotic hippocampus from TLE patients, but do not form functional GJ channels, most likely due to subcellular redistribution, morphological alterations and/or abnormal phosphorylation of Cx43.

## 4. Connexin Expression, Communication and Regulation in Experimental Epilepsy

The loss of astrocytic coupling observed in human tissue raises the question of whether this is causally involved in the genesis of epilepsy or whether it represents merely a consequence of the disease. To answer this question, information is required about the early pathophysiological changes that ultimately culminate in chronic epilepsy. As human tissue specimens are only available from the late chronic state of the disorder, animal models that closely mimic the human situation are needed. Most experimental TLE models involve the chemical or electrical induction of status epilepticus (SE) that triggers chronic epilepsy after a latent period of days to weeks [64,65,66]. Astrocyte changes occurring during SE or the latency period can be considered as possible causative factors in epileptogenesis. In accordance with human Cx expression studies, Cx43 and Cx30 mRNA and/or protein were found either unchanged or upregulated in rodent TLE models [12,67,68,69,70,71,72,73,74,75]. Intriguingly, in the unilateral intracortical kainate injection model of TLE-HS [24], the changes in astrocytic Cx expression, subcellular distribution and phosphorylation closely resembled those described in human epileptic tissues. Consistently, of the two astrocytic Cxs, only total protein levels of Cx43 were upregulated in the sclerotic hippocampus, while the membrane-associated levels were unaltered. Also in accordance with the situation in human tissue, Cx43 exhibited subcellular redistribution towards perivascular endfeet and an altered phosphorylation pattern in HS [12]. Interestingly, the model also recapitulated the complete loss of GJ coupling between astrocytes in chronic human TLE and revealed that uncoupling and the associated disruption of K^+^ buffering started at a very early stage of epileptogenesis, even before the onset of neurodegeneration and chronic seizure activity [24]. This finding implies that the uncoupling of astrocytes plays a causal role in the initiation of TLE. A similar reduction in GJ coupling was also observed 5 days after hyperthermia-induced febrile seizures [76], a finding that might provide a mechanistic explanation for the increased risk of developing human TLE following prolonged juvenile febrile seizures [77]. In contrast, Takahashi and colleagues reported increased coupling between hippocampal astrocytes one week after systemic injection of kainate into rats [74]. Differences between models (e.g., in the time course of histopathological and functional changes associated with epileptogenesis) may account for these contradictory findings.

The signalling pathway underlying loss of astrocyte coupling in TLE remains unknown, but there are good reasons to assume that increased levels of pro-inflammatory cytokines are involved. Indeed, inflammation is a hallmark of both human and experimental epilepsy, and there is evidence that the process is not only a consequence but also a causal factor in the aetiology of the disease [78]. Initial evidence for the regulation of astrocytic GJ coupling by inflammatory mediators came from cell culture studies, which showed that the pro-inflammatory cytokines interleukin-1β (IL-1β) and tumour necrosis factor α (TNFα) released from microglia inhibit coupling between cultured astrocytes [79,80,81]. Interestingly, the cytokines exerted an opposite effect on the Cx HCs, which were activated in their presence [80]. In later work, inhibition of astrocytic coupling could also be induced in situ after incubation of acute hippocampal slices from mice with IL-1β and TNFα, as well as in vivo by means of systemic injection of the inflammation-inducing molecule lipopolysaccharide (LPS) [24]. Direct demonstration of the involvement of microglia-derived cytokines in astrocytic uncoupling in epileptic tissue was recently provided by Henning and co-workers, who showed that seizure activity triggered by intracortical kainate injections decreased GJ coupling between hippocampal astrocytes only in acute slices from wild-type mice, but not in slices from transgenic mice with microglia-specific TNFα deletion or from mice with depleted microglia [82]. These data suggest that at this early time point (4 h after kainate injection), astrocytic coupling is inhibited by microglial TNFα. The cytokine probably exerts its effects through binding to astrocytic TNF receptors, which in turn might mediate the observed changes in Cx expression and/or phosphorylation [12,76] or even trigger necroptotic astrocytic cell death as recently described in the same model [83]. Depletion of microglia attenuated the severity of SE [82], a finding that is in line with the proposed seizure-promoting role of astrocytic uncoupling.

Another pathological mechanism that may be involved in GJ regulation arises from the fact that epilepsy is associated with a breakdown of the BBB, allowing serum proteins such as albumin to enter the brain parenchyma [84]. Extravasated serum albumin was proposed to be endocytosed by astrocytes via binding to transforming growth factor beta receptors (TGFβR) to reduce Cx expression and GJ coupling [85,86,87]. However, in a recent study, no major astrocytic albumin uptake could be detected 4 h and 1 d after intracortical kainate injection, and inhibition of TGFβR1 kinase did not prevent seizure-induced GJ uncoupling, arguing against an involvement of this mechanism in the regulation of GJs during early epileptogenesis [88]. Nevertheless, as uncoupling and albumin extravasation was observed at the chronic stage in this TLE model [12,24], a later contribution of the albumin-TGFβR1 signalling pathway to astrocytic dysfunction cannot be ruled out.

## 5. Impact of Glial Cx Gene Knockout on Neuronal Excitability and Epileptogenesis

Important insights into the role of astrocytic GJ channels in the regulation of neuronal excitability and susceptibility to seizures have been gained from transgenic mice with coupling-deficient astrocytes, which were generated by Wallraff and colleagues via crossing astrocyte-specific Cx43-deficient mice (Cx43^fl/fl^:*hGFAP*-Cre mice [89]) with mice globally lacking Cx30 (Cx30^−/−^ [30,90]). The resulting double knockout (dKO) mice showed not only a complete disruption of astrocytic coupling and, as mentioned above, impaired K^+^ and glutamate buffering, but also spontaneous epileptiform activity in acute hippocampal slices [30]. In a subsequent study, dKO mice exhibited a higher number of but less severe pentylenetetrazol-induced acute seizures in vivo during short-term (30 min) electroencephalogram (EEG)/video recording, a finding the authors attributed to increased neuronal excitability but decreased neuronal release probability and synchronisation [91]. Possibly, the lower energy supply due to lack of GJ coupling accounted for less severe seizures. Recently, dKO mice were subjected to the intracortical kainate injection model of TLE-HS to assess not only the consequences of Cx deficiency on seizure susceptibility but also on the whole process of epileptogenesis [92]. Remarkably, dKO mice showed substantially increased seizure and interictal spike activity during the chronic phase but less pronounced HS, indicating that the interruption of astrocytic GJ communication promotes chronic seizures but attenuates seizure-induced histopathological changes [92]. However, neither the constitutive dKO mice nor the recently generated inducible, astrocyte-specific dKO mice (Cx30^fl/fl^:Cx43^fl/fl^:GLAST^CreERT2^; [40]) showed spontaneous seizures or abnormal EEG activity in vivo [40,91]. Of course, one must bear in mind that in dKO mice, not only intercellular coupling but also the Cx proteins are missing, which does not correspond to the situation in human and experimental epilepsy where the proteins are even upregulated [12]. Hence, potential pro-epileptic non-channel and HC functions of Cxs are not considered in these mice. Moreover, astrocyte uncoupling in human and experimental TLE is spatially restricted to the sclerotic area and the epileptic foci, while Cx deletion in dKO mice involves the entire brain. Finally, compensatory developmental changes in the constitutive mouse line and residual coupling due to incomplete Cre recombination in the inducible mouse line limit the conclusions that can be drawn from these experiments.

Mice lacking only Cx30 developed less severe behavioural convulsions during the first 2 h after systemic kainate injection, a phenomenon that was dependent only on the presence of the protein but not on functional channels [72]. The authors attributed this to more effective astrocytic glutamate clearance in the absence of Cx30, which is consistent with the above-mentioned non-channel function of the protein in controlling the invasion of astrocytic processes into the synaptic cleft [51,72].

## 6. Therapeutic Potential of Targeting Glial Cx Channels in Epilepsy

The low efficacy and tolerability of currently available AEDs and the lack of antiepileptogenic, disease-modifying drugs underscore the urgent need for new cellular and molecular targets and therapeutic strategies in drug development. The evidence from the literature described above suggests that Cx channels, especially those composed of Cx43, play an important role in epilepsy and may therefore constitute promising targets for new AEDs. Interestingly, it has been shown that impaired GJ coupling in cytokine- or LPS-incubated astroglia/microglia co-cultures or in acute hippocampal slices from LPS-injected mice could be improved through treatment with the commonly used AED levetiracetam, leading to the assumption that the antiepileptogenic properties of the drug are at least partly attributable to its effect on coupling [24,81]. The suitability of substances that directly inhibit or increase GJ coupling as antiepileptic drugs is questionable, as Cxs are ubiquitously expressed in almost all tissues and cell types [93] and, therefore, systemic administration likely has side effects. For example, Cx43 is the most abundant GJ protein in the heart, where it plays a key role in the coordinated spread of electrical activity. Thus, its inhibition or activation in the heart would have deleterious effects on heart function. Another problem is the lack of specificity of substances that modulate GJs. For instance, carbenoxolone (CBX), the most commonly used GJ blocker, is not only unable to distinguish between different Cx isoforms or between GJ channels and HCs, but was also shown to inhibit voltage-gated Ca^2+^ channels, purinergic P2X7 receptors and tonic GABA_A_ receptor currents [94,95,96]. Accordingly, several studies have shown that CBX inhibits synaptic transmission and neuronal network excitability in a GJ-independent manner, questioning its suitability for assessing the role of GJs in epilepsy [94,97,98,99,100]. This should be kept in mind when drawing conclusions from the extensive literature on antiepileptic effects of CBX treatment in epilepsy models (for review see [101]). Similarly, no substances are known to specifically increase astrocytic coupling. Regarding systemic side effects, it would probably make more sense not to search for more specific GJ blockers/activators but instead to search for substances that prevent/reverse the molecular mechanisms or signalling pathways that lead to impaired coupling under pathological conditions. For example, it was recently reported that epileptiform activity inhibits astrocytic coupling via Na^+^/HCO3^−^ cotransporter-mediated intracellular alkalization and that blockers of this cotransporter have antiepileptic effects [102]. In this context, polyamines such as spermine have been shown to accumulate in astrocytes and to prevent or reverse the proton- or calcium-mediated inhibition of Cx43 [103,104]. As data from human and experimental TLE indicate that GJ inhibition is mediated by phosphorylation-mediated closure of Cx43 channels [12,76], compounds that disrupt aberrant Cx phosphorylation may have therapeutic potential in the treatment of epilepsy. In this regard, antiarrhythmic peptides such as the Cx43-targeting peptide danegaptide (ZP1609), developed for the treatment of reperfusion injury after acute myocardial infarction [105], were investigated [8,46]. Indeed, danegaptide was found to normalize pathological phosphorylation-induced closure of Cx43 channels not only in the heart, but also between astrocytes in a mouse brain ischemia/reperfusion model [106,107]. However, the peptide exhibited no effect on astrocyte uncoupling induced by intracortical kainate injections (unpublished data from our group), indicating that the mechanism of Cx43 inhibition differs between epilepsy and ischemia.

In addition to GJ channels, specific Cx HC blockade has also been proposed as a promising strategy for treating epilepsy [45,47]. In accordance with the proposed seizure-promoting consequences of HC opening, inhibition of Cx43 HCs with the Cx mimetic peptide TAT-Gap19 or the small molecule D4, which were described to block HC activity without affecting GJ coupling, yielded anticonvulsant effects in mice and rats in different models of seizures and epilepsy [45,47]. The advantage of this approach is that HC activation occurs only under pathological conditions, so systemic administration of HC blockers should not have major side effects [45,47]. However, more research is needed to understand the biophysical properties and specific role of these channels in the healthy and diseased brain before the therapeutic potential of Cx HC blockers can be established.

## 7. Conclusions

Evidence is accumulating showing that astrocyte Cx channels are critically implicated in epilepsy, but the underlying mechanisms and therapeutic importance are still poorly understood. In the sclerotic hippocampus of TLE patients, astrocytic GJ coupling is completely lost, despite the fact that expression of the two astrocytic Cx isoforms does not decrease. This might simply be due to loss of physical contact between glial processes as a result of the dramatic morphological alterations astrocytes undergo in sclerosis (Hinterkeuser et al., 2000; Bedner et al., 2015) [24,62]. However, it can also not be excluded that besides altered intercellular GJ coupling, increased Cx HC activity or non-channel properties of Cxs affect neuronal excitability and synchronisation. Maybe, only a combination of several of these factors is sufficient to generate neuronal hyperexcitability, which would explain why mice with genetic deletion of astrocytic Cxs do not exhibit spontaneous behavioural convulsions. Identifying the exact mechanisms underlying generation and progression of this complex disease remains a challenge. Nevertheless, since astrocytic uncoupling is one of the earliest detectable cellular changes in experimental TLE that precedes the onset of neurodegeneration and chronic seizures, it can be considered as a causative factor. To validate the causality of the dysfunction, it would be necessary to prove that its suppression prevents the genesis of the disease. However, this requires detailed knowledge of the mechanism of uncoupling and the identification of substances that specifically inhibit it. The growing recognition of the importance of the astrocytic network in epilepsy, together with the increasingly sophisticated technical tools and methods available today, give rise to optimism that the mechanisms governing epilepsy will soon be better understood. This opens new avenues for future research and therapeutic interventions.

## Data Availability

Data sharing not applicable.

## Abbreviations

AED: antiepileptic drug, BBB: blood–brain barrier, CBX: carbenoxolone, Cx: connexin, Cx30: connexin30, Cx43: connexin43, dKO: double knockout, EEG: electroencephalogram, GJ: gap junction, HC: hemichannel, HS: hippocampal sclerosis, [K+]o: extracellular K+ concentration, IL-1β: interleukin-1β, LPS: lipopolysaccharide, SE: status epilepticus, TGFβR: transforming growth factor beta receptor, TLE: temporal lobe epilepsy, TNFα: tumor necrosis factor α.
